# Beyond protein-coding genes

**DOI:** 10.7554/eLife.45123

**Published:** 2019-02-19

**Authors:** Anna Lozano-Ureña, Sacri R Ferrón

**Affiliations:** 1 Department of Cell BiologyUniversity of ValenciaValenciaSpain; 2 ERI BiotecMedUniversity of ValenciaValenciaSpain

**Keywords:** long non-coding RNA, intellectual disabilities, autism, genomics, gene regulation, neuronal development, Human, Mouse

## Abstract

A long non-coding RNA called *lnc-NR2F1* regulates several neuronal genes, including some involved in autism and intellectual disabilities.

**Related research article** Ang CE, Ma Q, Wapinski OL, Fan S, Flynn RA, Lee QY, Coe B, Onoguchi M, Olmos VH, Do BT, Dukes-Rimsky L, Xu J, Tanabe K, Wang L, Elling U, Penninger JM, Zhao Y, Qu K, Eichler EE, Srivastava A, Wernig M, Chang HY. 2019. The novel lncRNA *lnc-NR2F1* is pro-neurogenic and mutated in human neurodevelopmental disorders. *eLife* **8**:e41770 . doi: 10.7554/eLife.41770

Most of the mammalian genome does not encode for working proteins. However, much of this non-coding DNA is still transcribed, often to produce RNA products that have a role in development. Some of these molecules are called long non-coding RNAs (lncRNAs) because they contain more than 200 base pairs: these transcripts fine-tune gene expression by interacting with chromatin and the transcription machinery inside cells (Ponting et al., 2009). The brain expresses more lncRNAs than any other part of the body (Derrien et al., 2012), but we know relatively little about the roles these molecules play in this organ (D'haene et al., 2016; Aprea et al., 2013). Learning more about lncRNAs will be essential if we are to understand both the typical and the atypical brain.

In intellectual disabilities or autism spectrum disorders, defects in cognitive abilities, such as social interaction and communication, can appear early in development and persist into adulthood. These neurodevelopmental disorders involve abnormal changes in the way genetic information is expressed (Rennert and Ziats, 2017). Several lncRNAs are associated with these conditions, sometimes being transcribed atypically (reviewed in van de Vondervoort et al., 2013). For instance, certain lncRNAs are expressed differently in patients on the autism spectrum (Ziats and Rennert, 2013).

Now, in eLife, Anand Srivastava, Marius Wernig, Howard Chang and colleagues – including Cheen Ang, Qing Ma and Orly Wapinski, all from Stanford University, as joint first authors – report that several lncRNAs that are involved in the formation of neurons are mutated or disrupted in children with autism spectrum disorder and intellectual disabilities (Ang et al., 2019).

Ang et al. started by reprogramming mouse cells called embryonic fibroblasts into neurons; this experiment helped them to identify 35 candidate lncRNAs that are both upregulated when neurons form and close to neuronal genes. Amongst those, 28 were present on the same chromosomes in humans and in mice. The group then tried to identify whether these 28 human candidates were mutated in disease by overlapping the lncRNAs sequences onto a map of mutations found in children with neurodevelopmental disorders and congenital defects. This analysis highlighted five lncRNAs that were often mutated in affected individuals, and which happened to also be expressed during human brain development. One of them, called *lnc-NR2F1*, was adjacent to *NR2F*, a gene which encodes a transcription factor that helps neurons form and wire together (Borello et al., 2014).

The team, which is based at Stanford, Clemson University, the University of Washington, the Greenwood Genetic Center, and the Austrian Academy of Sciences, found patients with developmental delays who expressed normal levels of the coding *NR2F1* gene but presented a unique disruption of the *lnc-NR2F1* gene. Most of the brain lncRNAs are located near genes that code for proteins, and it is believed that both lncRNAs and protein-coding genes are expressed at the same time (Ponjavic et al., 2009). However, the work by Ang et al. potentially indicates that *lnc-NR2F1*, rather than *NR2F1*, might contribute to the clinical symptoms associated with neurodevelopmental disorders. If so, this would strengthen the hypothesis that lncRNAs are independent transcriptional units that activate gene expression in the brain.

Then, Ang et al. discovered that, in mouse cells, *lnc-Nr2f1* enhanced the transcription of genes that create and guide the structures which allow neurons to connect. Deleting or overexpressing *lnc-Nr2f1* changed how these genes were expressed, and how the cells looked and worked. In addition, *lnc-Nr2f1* was shown to attach to the genes, suggesting that it binds chromatin to regulate gene expression (Figure 1). While we still do not fully understand the physiological changes that accompany neurodevelopmental disorders, the results by Ang et al. suggest that lncRNAs themselves may contribute to these conditions, or that they drive the expression of disease-associated genes.

**Figure 1.**
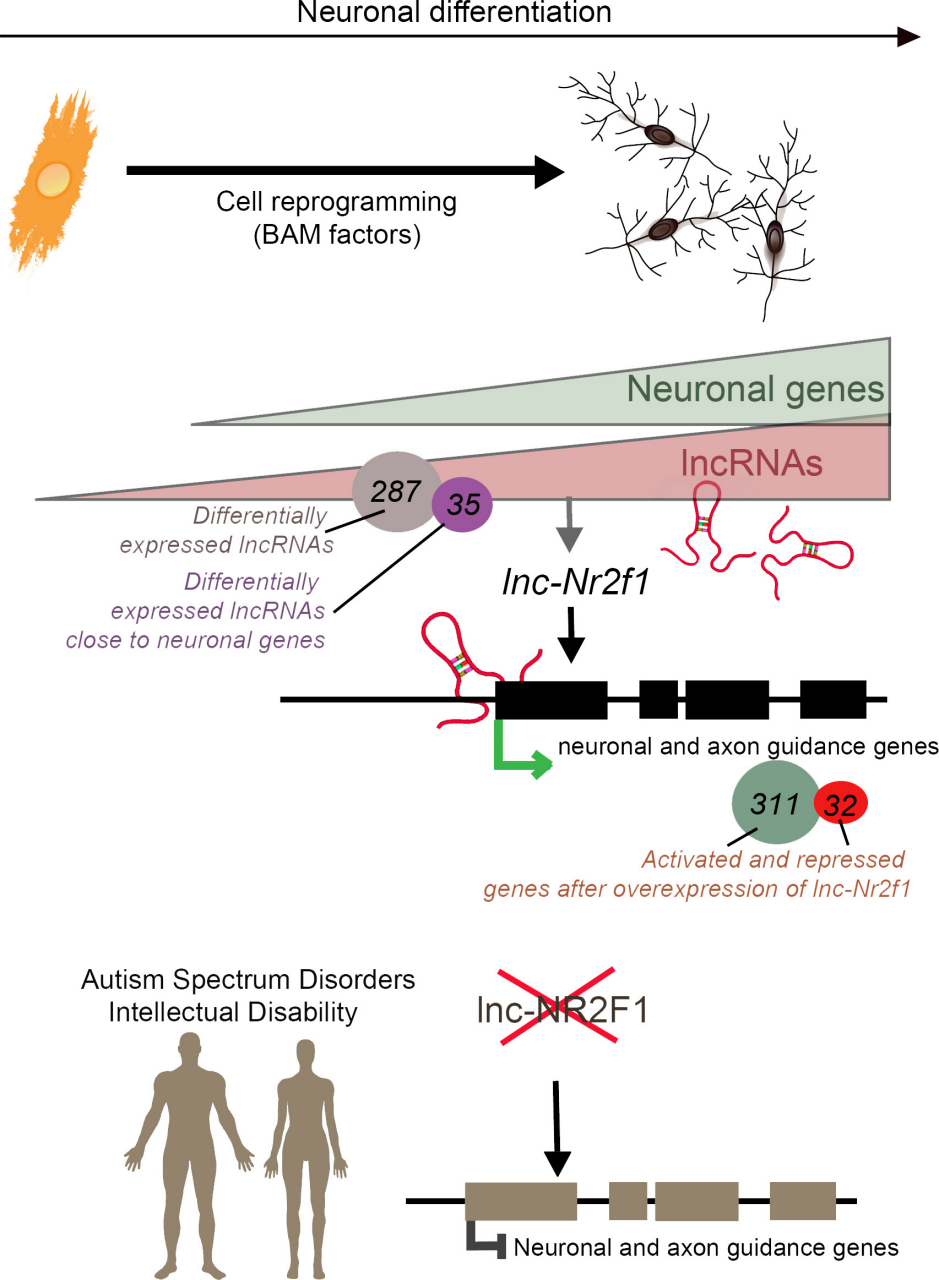
Long non-coding RNAs (lncRNAs) and neuronal development in neurodevelopmental disorders. Mouse embryonic fibroblasts (orange) were reprogrammed into neurons (top right) using transcription factors called BAM factors. This led to an increase in the expression of neuronal genes (green triangle) and lncRNAs (pink triangle). Of the 287 lncRNAs that were differentially expressed, 35 were close to neuronal genes. One of these, *lnc-Nr2f1* (red loops), binds to mouse neuronal and axon guidance genes (black boxes) and promotes their transcription (green arrow). The overexpression of *lnc-Nr2f1* resulted in 311 neuronal genes being upregulated and 32 being repressed. The expression of *lnc-NR2F1* is altered (red cross) in patients with autism spectrum disorders and intellectual disabilities, and this potentially disrupts the transcription of human neuronal and axon guidance genes (brown boxes; black inhibitory arrow). It is therefore possible that *lnc-NR2F1* is involved in these conditions.

How mutations in protein-coding genes contribute to disease is widely studied, yet most mutations are found in regions that do not code for proteins. Understanding how lncRNAs regulate genes during brain development provides a way to tie genetic variation with changes in gene expression in neurodevelopmental disorders. Building on the findings by Ang et al., it may be possible to examine how clinical phenotypes, cellular responses and lncRNAs are connected in these conditions, potential unearthing new targets for therapeutic intervention.
